# Thermodynamic Characteristics of Phenacetin in Solid State and Saturated Solutions in Several Neat and Binary Solvents

**DOI:** 10.3390/molecules26134078

**Published:** 2021-07-03

**Authors:** Maciej Przybyłek, Anna Kowalska, Natalia Tymorek, Tomasz Dziaman, Piotr Cysewski

**Affiliations:** 1 Department of Physical Chemistry, Pharmacy Faculty, Collegium Medicum of Bydgoszcz, Nicolaus Copernicus University in Toruń, Kurpińskiego 5, 85-950 Bydgoszcz, Poland; m.przybylek@cm.umk.pl (M.P.); 288310@stud.umk.pl (A.K.); 288369@stud.umk.pl (N.T.); 2 Department of Clinical Biochemistry, Pharmacy Faculty, Collegium Medicum of Bydgoszcz, Nicolaus Copernicus University in Toruń, Karłowicza 24, 85-950 Bydgoszcz, Poland; tomekd@cm.umk.pl

**Keywords:** phenacetin, fusion thermodynamics, ideal solubility, heat capacity, excess solubility, synergistic effect, co-solvency

## Abstract

The thermodynamic properties of phenacetin in solid state and in saturated conditions in neat and binary solvents were characterized based on differential scanning calorimetry and spectroscopic solubility measurements. The temperature-related heat capacity values measured for both the solid and melt states were provided and used for precise determination of the values for ideal solubility, fusion thermodynamic functions, and activity coefficients in the studied solutions. Factors affecting the accuracy of these values were discussed in terms of various models of specific heat capacity difference for phenacetin in crystal and super-cooled liquid states. It was concluded that different properties have varying sensitivity in relation to the accuracy of heat capacity values. The values of temperature-related excess solubility in aqueous binary mixtures were interpreted using the Jouyban–Acree solubility equation for aqueous binary mixtures of methanol, DMSO, DMF, 1,4-dioxane, and acetonitrile. All binary solvent systems studied exhibited strong positive non-ideal deviations from an algebraic rule of mixing. Additionally, an interesting co-solvency phenomenon was observed with phenacetin solubility in aqueous mixtures with acetonitrile or 1,4-dioxane. The remaining three solvents acted as strong co-solvents.

## 1. Introduction

Phenacetin (CAS: 62-44-2, IUPAC name: *N*-(4-ethoxyphenyl)acetamide) is a white crystalline odorless substance. This organic compound is an acetanilide derivative and a close analog of paracetamol, with the hydroxyl group replaced with an ethanolic group. It was introduced as a drug in the late nineteenth century, and exhibits analgesic and antipyretic activities due to its cyclooxygenase-3 inhibiting properties [1,2]. However, its use as a pain treatment has been limited due to its many side effects, including methemoglobinemia and hemolytic anemia [3,4,5], and its potential carcinogenic properties [6,7]. In general, phenacetin is considered a poorly water-soluble drug, and for this reason it has been the subject of several studies aimed at improving solubility and dissolution rates, including drug-polymer dispersions [8], micronisation [9] and recrystallization from surfactants solutions [10]. Conversely, phenacetin is considered as soluble in acetone and pyrimidine [11], which are typical proton-acceptor solvents.

Solubility is one of the most important properties characterizing pharmaceuticals, and analgesic and anti-inflammatory drugs are frequent subjects of research on solubility improvement techniques such as nanocrystals [12] or co-crystals and salts preparation [13]. Many theoretical models were formulated for the interpretation of temperature-related solubility as well as solid-liquid equilibrium phase diagrams. Some of the most popular models are van’t Hoff [14], Apelblat [15,16], Buchowski–Ksiazczak (λh) [17], Wilson [18], NRTL [19], and Jouyban–Acree [20]. These approaches became popular due to the quality of data fitting, model simplicity, and a relatively small number of variables [21,22,23,24,25,26]. These empirical or semi-empirical approaches offer the thermodynamic interpretation of the dissolution process in relation to the concept of an ideal solubility, which is the amount of solute capable to be dissolved by an ideal solvent, i.e., the solvent characterized by activity coefficient equal to unity [27]. The ideal solubility value is the crystal-only property, which can be calculated if the fusion thermodynamic characteristics are available. Properties such as melting point and fusion enthalpy are quite often reported, however, the information on experimental heat capacity of solid and melt states is quite sparse. This is not only related to the fact that experiments are more sophisticated, but also to the commonly assumed notion that the more stable the crystal, the higher the melting point, the lower the enthalpy of fusion only, and these properties have a dominant contribution to fusion thermodynamics. Hence, ideal solubility is often inferred using only these properties and ignoring other contributions. However, there is compelling evidence that the relative value of the heat capacities of solid and melt states often has a non-trivial influence on the values of ideal solubility [28]. Another important characteristic offered by theoretical models is apparent thermodynamic calculation, which allows for estimating basic thermodynamic functions of solvation directly from the solubility data. Based on this approach, it can be assessed whether the dissolution is enthalpy or entropy-driven [29,30,31,32]. Notably, the knowledge of entropic/enthalpic contributions to the Gibbs free energy, as well as activity coefficients in saturated solutions analysis, is of a particular importance in describing the crystallization process [33,34].

Although some phenacetin solubility data in common neat and binary solvents have already been reported [35,36,37,38], the number of available datasets is insufficient. It is, therefore, worth expanding on the information available on dissolution of phenacetin in other media with a potential solubility enhancement. The aim of this study is threefold. Firstly, detailed knowledge of fusion thermodynamics is provided with measured temperature-related heat capacities. Secondly, the experimental pool of phenacetin solubility data is extended through the inclusion of several neat and aqueous-organic binary solvents not previously studied. Finally, comprehensive analysis of data is provided for the model of heat capacity change on melting and related properties such as fusion thermodynamics, ideal solubility, and activity coefficient in the solvents studied.

## 2. Results and Discussion

### 2.1. Thermochemical Analysis of Phenacetin

**Figure 1:**
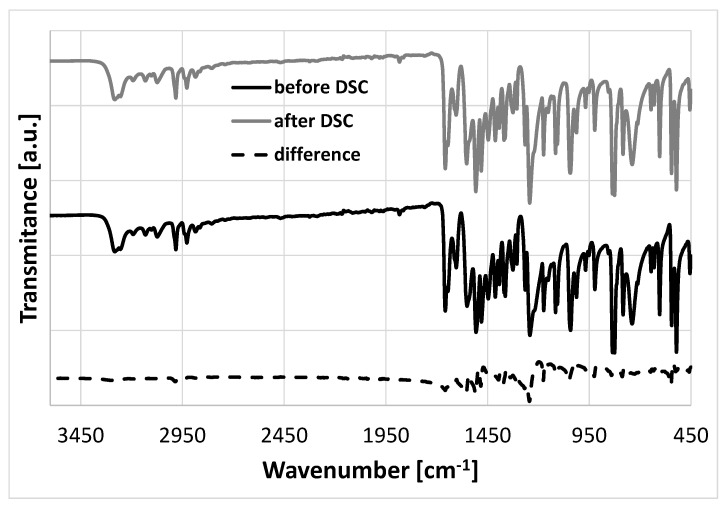
FTIR-ATR spectra recorded for phenacetin sample before and after DSC measurements.

Solid phenacetin adopts a monoclinic crystal structure which has been solved and deposited several times under common CSD ref code PYRAZB. No polymorphs or solvates have so far been reported. However, in order to confirm that no crystal phase or pseudo-polymorphic transitions occurred during solubility measurements, the sediments collected after flask-shake experiments were analyzed using DSC and FTIR-ATR techniques. The results of these measurements are summarized in supplementary materials in Figures S1 and S2. The solvate formation can be identified by the absorption band shifts on the IR spectra. With DSC thermograms, the formation of a new crystal form would be associated with polymorphic transformations or solvate degradation found prior to the melting peak. Both IR spectra and DSC thermograms recorded for sediments were similar to pure phenacetin. Furthermore, to confirm that phenacetin did not degrade during DSC measurements and the same chemical structure was preserved, the IR spectra of the sample was measured before and after the entire DSC cycle. As documented in Figure 1, both spectra are nearly identical as indicated by the differential plot. Furthermore, the samples were weighed before and after each measurement and no weight loss was found, additionally confirming that the samples did not degrade and did not sublimate during the calorimetric measurement.

**Figure 2:**
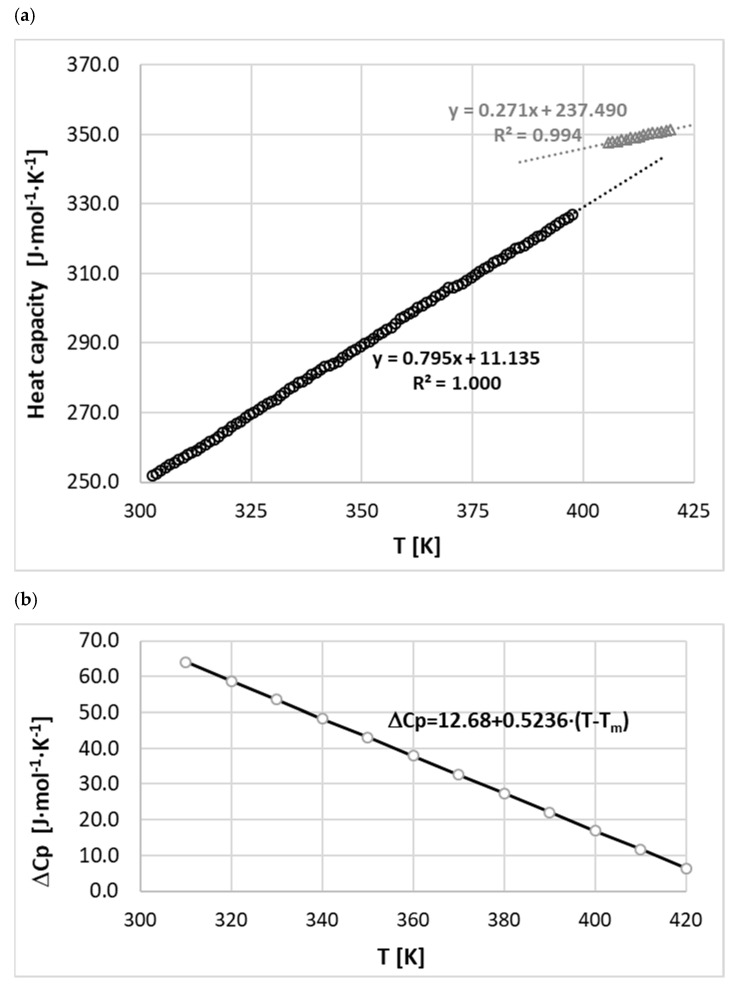
Distributions of measured values of heat capacities of phenacetin in solid and melt states (**a**), along with derived heat capacity change upon melting (**b**).

Phenacetin has previously been the subject of extensive thermochemical analysis, and data characterizing both temperature and heat of melting have been reported. This is summarized in Table 1, which also includes the values determined in this study and shows that our results are consistent with those previously reported. Since the values of heat capacity of solid and melt phenacetin have not previously been reported, the experimental thermochemical analysis was extended to include these characteristics. The values of heat capacities of phenacetin in both solid and melt states were measured in the broad temperature range documented in Figure 2. It is notable that the temperature trends are linear, and it is reasonable to expect that a change of heat capacity values associated with melting ∆Cp would adopt a linear temperature relationship. This is true in the case of phenacetin, as shown in Figure 2b. Phenacetin is stable across the range of temperatures used in this study, does not decompose, and does not undergo any changes.

### 2.2. Thermodynamics of Phenacetin Melting

The activity of phenacetin in saturated solutions is dependent on the pure solid activity due to imposed restrictions by chemical equilibrium conditions:

(1) $$\mu_{P}^{s}\left(T\right)=\mu_{P}^{sat}\left(T\right)$$

Consequently, the above constraint determines the values of the mole fraction of the solute:

(2) $$lnx_{eq}=lna_{eq}-ln\gamma_{eq}$$

This in turn allows for quantification of the values of the activity coefficients $\gamma_{eq}$ as the measure of deviation from the ideal system, provided that the ideal solubility is computed. This quantity is directly computable based on solid activity in relation to fusion Gibbs free energy $\Delta G_{}^{fus}\left(T\right)$:

(3) $$lna^{s}=-\frac{\Delta G^{fus}}{RT}$$

The Gibbs free energy is related to the enthalpic ($\Delta H_{}^{fus}\left(T\right))$ and entropic ($T\cdot \Delta S_{}^{fus}\left(T\right)$) contributions according to the well-known thermodynamic relationship:

(4) $$\Delta G_{}^{fus}\left(T\right)=\Delta H_{}^{fus}\left(T\right)-T\cdot \Delta S_{}^{fus}\left(T\right)$$

The experimental characteristics determined by calorimetric techniques are necessary for solving the above equations. It is worth distinguishing melting from fusion. The former denoted herein by subscript “*m*” is restricted to fusion at melting point. The phase change at other conditions is referred as fusion, and the effect of the temperature of fusion thermodynamic functions can be described using Kirchhoff’s law:

(5) $$\Delta H_{}^{fus}=\Delta H_{}^{fus}\left(T_{m}\right)+\overset{T}{\underset{T_{m}}{\int }}\Delta C_{p}dT$$

(6) $$\Delta S_{}^{fus}=\frac{\Delta H_{}^{fus}\left(T_{m}\right)}{T_{m}}+\overset{T}{\underset{T_{m}}{\int }}\frac{\Delta C_{p}}{T}dT$$

where heat capacity change upon melting is the difference between liquid and crystal states:

(7) $$\Delta C_{p}^{fus}\left(T\right)=C_{p}\left(l\right)\left(T\right)-C_{p}\left(s\right)\left(T\right)$$

This value is often represented by the following linear form [48,49,50]:

(8) $$\Delta C_{p}\left(T\right)=q+r\left(T_{m}-T\right)$$

This mathematical function reflects the assumption that the extrapolation of Δ*Cp*(*T*) adopts a linear relationship with two constant parameters, where values are typically fitted to experimental data for measured values of heat capacity. By combining the above relationships one can be obtain the following directly applicable set of equations:

(9) $$\Delta H^{fus}=\Delta H^{m}\left(T_{m}^{}\right)+q\left(T-T_{m}^{}\right)-\frac{r}{2}{\left(T-T_{m}^{}\right)}^{2}$$

(10) $$\Delta S^{fus}=\frac{\Delta H^{m}\left(T_{m}^{}\right)}{T_{m}^{}}+q\cdot ln\frac{T}{T_{m}^{}}+r\left(T_{m}^{}ln\frac{T}{T_{m}^{}}-T+T_{m}^{}\right)$$

Since no polymorphic variation is observed in the case of phenacetin, there is no need to account for contributions originating from phase transitions. Using the relationships defined in Equations (4), (9) and (10), full fusion thermodynamics can be characterized if parameters *q* and *r* are measured. Additionally, in the case of the ideal solution and setting the activity coefficient of solute in the solution to unity, the above relationships provide a direct means of computing ideal mole fraction solubility:

(11) $$lnx_{1}^{id}=\frac{\Delta H_{}^{m}}{R}\left(\frac{1}{T_{m}}-\frac{1}{T}\right)-\frac{1}{RT}\int_{T_{m}}^{T}\Delta C_{p}dT+\frac{1}{R}\int_{T_{m}}^{T}\frac{\Delta C_{p}}{T}dT$$

**Figure 3:**
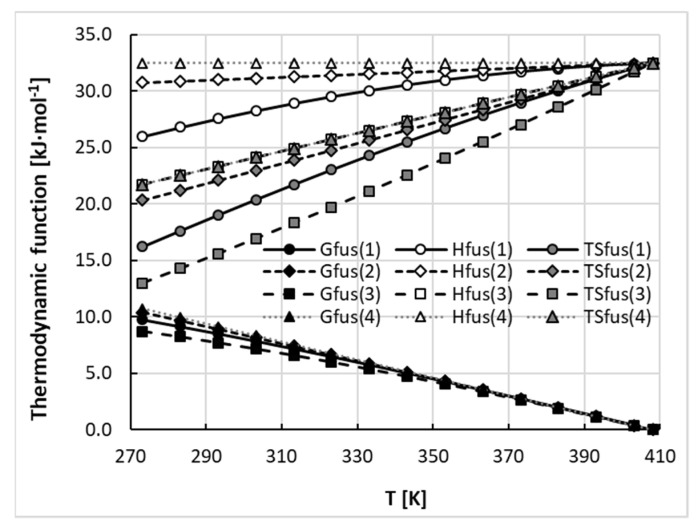
Thermodynamic properties of phenacetin according to the different assumptions for heat capacity change upon melting defined in Table 2.

**Figure 4:**
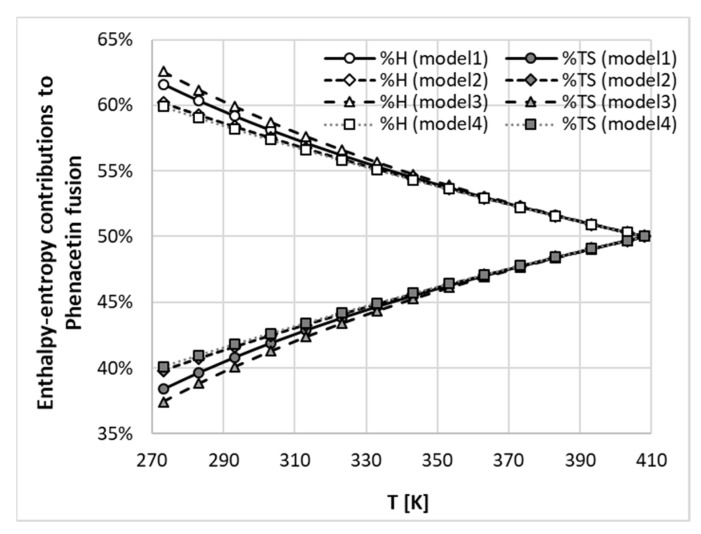
The temperature trends of enthalpy and entropy contributions to fusion Gibbs free energy expressed as weighted percentages %*H* = |∆*X^fus^*|/(|∆*H^fus^*| + |∆*TS^fus^*|), where *X* = *H* or *TS*.

Measurement of heat capacity change on melting is difficult, and there are some suggested approximations, but their relevance is still debatable [28,51,52]. Often these simplifications are necessitated by such properties as sublimation or degradation of solids below melting point. It is clear that models of ∆*Cp* affect the computed values of ideal solubility and consequently the activity coefficients. In many applications [52,53,54,55,56,57,58] the heat capacity change-containing terms in Equation (11) are deemed to be negligible and can be ignored. This relies on the observation that absolute values of molar enthalpy of fusion are much higher when comparing two other terms which, having opposite signs, may cancel each other. This approximation can be defined by setting to zero the values of both parameters in Equation (8). Although this is the crudest of simplifications, there are results in the literature which suggest that this approximation is generally valid in conditions typical for the processing of organic substances [28,51,52]. A further approach does not completely ignore the values of ∆*Cp*(*T*) but assumes them to be temperature-independent and approximately equal to the values of melting entropy [59]. Again, there are proponents and opponents of such an approach as already discussed [60]. There is also the possibility of replacing melting entropy with the measured value of heat capacity change at melting temperature. These four approaches, including one fully accounting for ∆*Cp*(*T*), are summarized in Table 2, and they were applied to the detailed analysis of fusion thermodynamics of phenacetin. The results achieved are presented in Figure 3 and reveal a surprising conclusion. It can be directly inferred from the plots shown that, in the case of this drug, the temperature variation of fusion Gibbs free energy (denoted by black filled symbols) is very similar for all four models, despite significant changes in the values of fusion enthalpy (grey symbols) and entropy (open symbols) with temperature alterations. What is even more surprising is that the entropy-enthalpy compensation of the fusion process is also very similar. These two aspects of the fusion mechanism are the measures of the driving forces associated with phase change of pure solids. In Figure 4 these contributions are presented by plotting weighted percentages calculated as follows:

(12) $$\%X=\frac{\left|\Delta X^{fus}\right|}{\left|\Delta H^{fus}\right|+\left|T\Delta S^{fus}\right|}$$

where *X* stands for *H* or *TS*. From Figure 4 it can be concluded that, in the full range of temperatures, the enthalpy contribution dominates over the entropy contribution up to melting point. The conclusion drawn from the data presented in Figure 3 and Figure 4 is that, from the perspective of the fusion thermodynamics of phenacetin, the accuracy of the model for heat capacity change upon melting is of secondary importance, and even the crudest simplification offers an acceptable estimation of the values of fusion Gibbs free energies and fusion enthalpy-entropy compensation.

### 2.3. Ideal Solubility of Phenacetin

**Figure 5:**
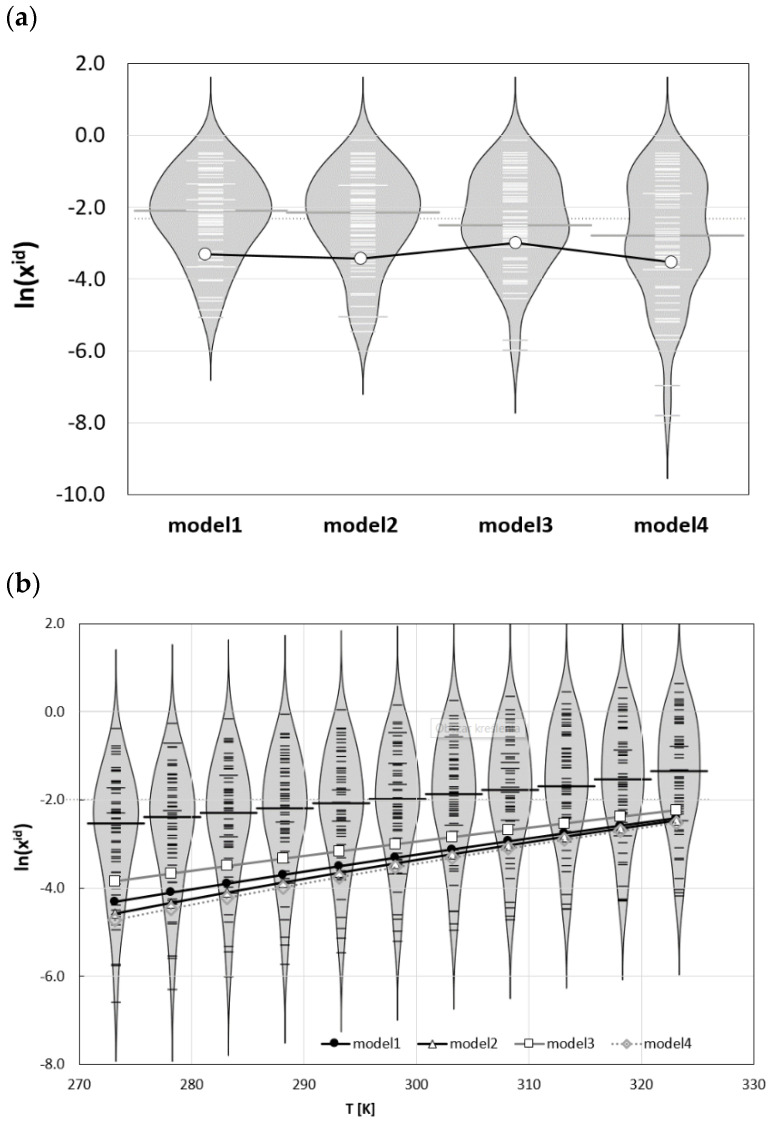
Distribution of phenacetin ideal solubility computed using different models of ∆*Cp*(*T*), overlaid over (**a**) bean plots of ideal solubility of 60 selected solids computed using different models of ∆*Cp*(*T*) for ambient conditions, and (**b**) as a function of temperature using model 1.

**Figure 6:**
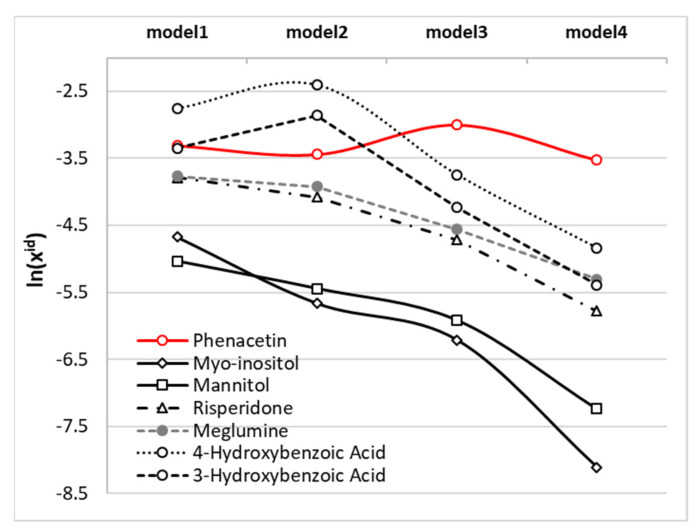
Distribution of ideal solubility computed at room temperature using different models of ∆*Cp* for exemplary solids. The literature experimental data used for calculations were obtained from ref. [61] (myo-inositol, mannitol), ref. [62] (risperidone), ref. [50] (meglumine), and ref. [48] (4-hydroxybenzoic acid, 3-hydroxybenzoic acid).

The influence of the ∆*Cp*(*T*) model on the values of ideal solubility is also of interest, and these results are presented in Figure 5, along with distributions of temperature-related ideal solubility computed for the set of 60 selected solids. Since models 1 and 2 required the experimental heat capacities data, only solids with available data were included in the analysis. Compared to solubility data counted in thousands of solutes, there is significantly less data relating to full thermodynamic characteristics. The set used here serves as a reference point for reviewing the influence of the analysed models on phenacetin properties in the broader perspective and with adequate scales. In Figure 5a the lines represent phenacetin ideal solubility computed at room temperature and bean plots characterize the reference set. A bean plot is a means of visually comparing distributions of numeric data, where the shape represents the data density, and the short horizontal lines denote data points. The beans plotted in Figure 5a allow for comparison of the distributions of ideal solubility obtained using the four analysed models of ∆*Cp*. Dark grey lines on each bean represent the median value of each batch distribution. This value is slightly reduced with progression of the ∆*Cp* model simplification from −2.16 down to −2.88 for models 1 and 4 respectively. The value characterizing ideal solubility of phenacetin is smaller compared to median irrespective of the model applied. This suggests a lower tendency to fuse compared to median solid in the population and is comparable in this respect to 1,3,5-triphenylbenzene, dimethyl terephthalate, or erythritol. The differences between computed values of ideal solubility using different models are quite small and are equal to 14% (x^id^(2) = 0.032) and −3% (x^id^(4) = 0.029) if models 2 and 4 are compared with model 1, for which x^id^(1) = 0.037. Only in model 3 is a stronger deviation observed, reaching 63% (x^id^(3) = 0.050). Hence, the ideal solubility of phenacetin can be quite accurately obtained using the crudest approach and ignoring Δ*Cp* completely. This conclusion remains unchanged if temperature-related trends of ideal solubility are considered. In Figure 5b the plots corresponding to models 1 and 4 almost overlap. The overestimation of ideal solubility with the application of models 3 and 2 increases with decreasing temperature. The main conclusion drawn from the thermodynamic data of phenacetin is that an assumption of zero value of heat capacity change upon melting is surprisingly accurate in predicting ideal solubility. Since this observation is contrary to expectation, it was of interest to investigate whether this could be applied as a general rule, or if it should be applied to phenacetin only, and additional plots in Figure 6 characterise the ideal solubility for selected solids with high and positive ∆*Cp*(*T*) values. This comparison suggests that phenacetin is unique, and for all examples provided, the more restrictions imposed on the ∆*Cp*(*T*) model, the stronger the underestimation of ideal solubility observed. This suggests that level of approximation should be analysed separately for subjects of analysis, and generalization is not straightforward.

### 2.4. Solubility of Phenacetin in Neat Solvents

**Figure 7:**
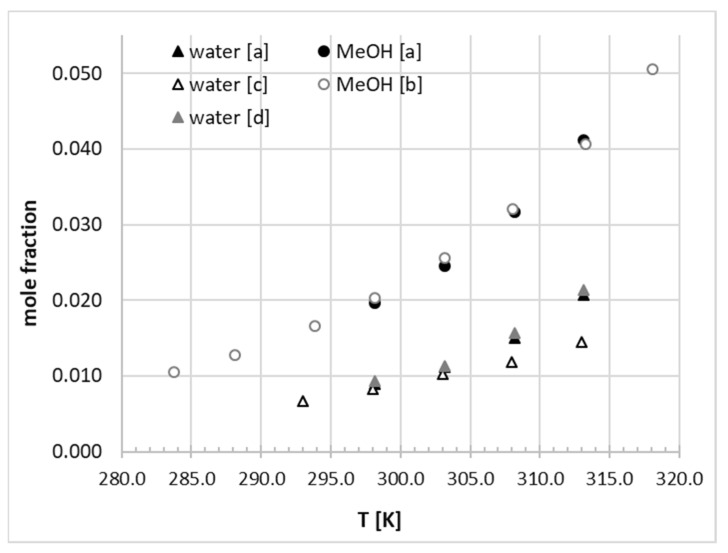
Comparison of selected phenacetin solubility measurements in neat solvents: [a] this study, [b] ref. [36], [c] ref. [63], and [d] ref. [35]. Values of water mole fractions were multiplied by a factor of 10^2^.

The measured temperature-related molar fractions of phenacetin determined in saturated solutions of six neat solvents (water, 1,4-dioxane, DMSO, DMF, acetonitrile, and methanol) are collected in Table 3. It can be seen that the solubility enhancement of the title compound can be ranked as follows: water < 1,4-dioxane (2.01) < acetonitrile (2.12) < methanol (2.34) < DMSO (2.58) < DMF (2.92) irrespective of the temperature. The solubility advantage (in parentheses) was quantified as the logarithm of solubility ratio with respect to water at room temperature. The low aqueous solubility of phenacetin was confirmed by previous studies as evidenced by the comparison provided in Figure 7. Notably, the available literature solubility data in water and methanol are consistent with the values obtained in this study. The highest hydrotropic effect was observed for highly polar aprotic solvents followed by polar-weak protic and weakly polar-aprotic solvents.

### 2.5. Solubility of Phenacetin in Aqueous Organic Solvent Binary Mixtures

**Figure 8:**
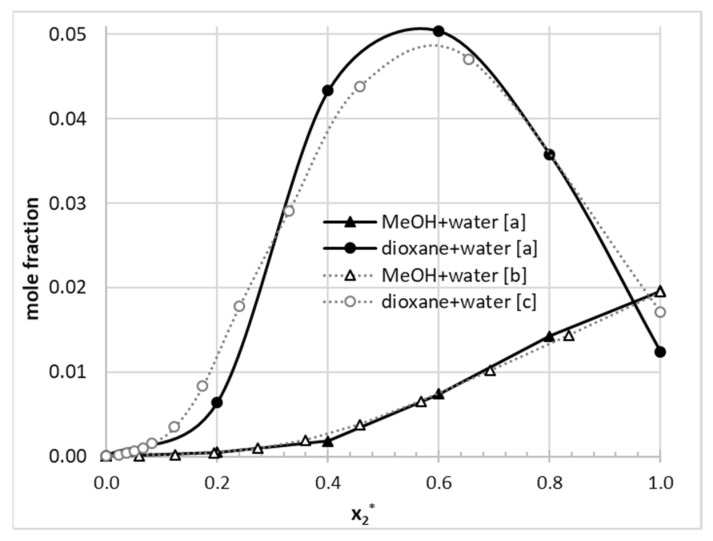
Comparison of solubility in binary solvents measured in [a] this study (*T* = 25 °C), with the literature data [b] ref. [37] (*T* = 25 °C), and [c] ref. [63] (*T* = 24.8 °C).

The data characterizing solvent effects on phenacetin solubility are shown in Table 4. The solubility values were determined for aqueous binary mixtures of acetonitrile, 1,4-dioxane, DMF, dimethylsulphoxide (DMSO), and methanol, at four temperatures—298.15, 303.15, 308.15, and 313.15 K. To illustrate graphically the effect of the binary solvent composition on solubility at different temperatures, the plotted trends are provided in supplementary materials in Figures S3–S7. A notable solubility advantage and synergistic effect was observed in relation to both pure solvents for acetonitrile-water and 1,4-dioxane-water systems. In the case of the former binary mixture, the highest phenacetin solubility was observed at *x*_2_* = 0.8 and was associated with a solubility advantage SA = 2.31 in respect of water SA = log(*X_cosolvent_*/*X_water_*). In the case of aqueous 1,4-dioxane mixture the synergistic effect occurred at *x*_2_* = 0.6, reaching 2.70 solubility advantage compared to water. In the case of the remaining three binary mixtures studied, a monotonous solubility enhancement is observed for the entire range of mole fractions of the organic components. It is worth mentioning that the experimental data measured in this study are consistent with available literature data as evidenced in Figure 8.

The interpretation of solubility in binary solvent mixtures can be performed using many alternative approaches, which differ greatly by concept and by underlying theoretical foundations. Among many available theoretical methods reviewed by Jouyban [64], two alternative classes can be distinguished. The first class of models interprets the complete set of solubility data in a given solvent mixture irrespective of temperature and solvent composition, and regression is performed using the values of global parameters characterizing the whole solute-solvent system. Many attempts have been made to extend the applicability of models originally developed for neat solvents and adapting them to describe multi-component systems. These models include a series of approaches taking advantage of Jouyban–Acree equations [20]. The second class of models can be exemplified by the van’t Hoff [14], Apelblat [15,16], Buchowski–Ksiazczak (λh) [17], Wilson [18], and NRTL [19] models. There are also combinations of these two classes of models which incorporate the second class of approaches into the Jouyban–Acree equation. As a result, these approaches utilize a varying number of parameters for experimental data. The main disadvantage of the second and third type of models is a sizable regression problem, which is typically a multiplication of the number of solvents ratios at given temperature by the number of parameters used in the model formulation. From the formal point of view, avoiding overfitting requires at least twice the number of data points than the number of parameters used for regression of experimental data. Since the solubility of phenacetin presented in this paper was measured at only four temperatures, the regression of such data should be performed with at most two parameters per system. That is why our solubility modelling was restricted to the first type of approaches in the simplest form. Interestingly, it enabled quantification of mutual solvents interference and allowed us to account for the non-additivity of solubility. This can be inferred from deviations of algebraic rule of mixing according to the following formula:

(13) $$ln\left(x_{1}^{bin}\left(T\right)\right)=\left(1-x_{2}\right)\cdot ln\left(x_{1}^{neat}\left(T,x_{2}=0\right)\right)+x_{2}\cdot ln\left(x_{1}^{neat}\left(T,x_{2}=1\right)\right)\;+\Delta^{exc}ln\left(x_{1}^{bin}\left(T\right)\right)$$

**Figure 9:**
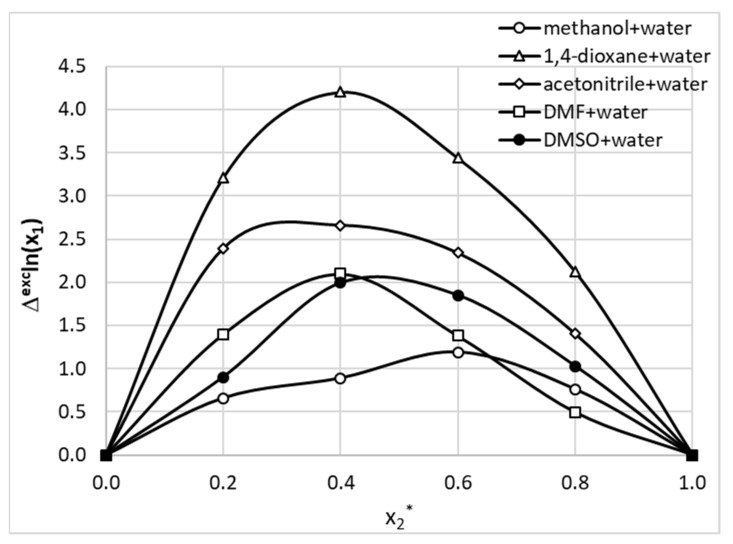
The trends of phenacetin excess solubility as a function of increasing value of organic solvent molar fraction in studied binary aqueous mixtures at room temperature.

The last term in Equation (13),$\;\Delta^{exc}ln\left(x_{1}^{bin}\left(T\right)\right)$, accounts for non-additivity of solubility in binary mixture with respect to neat solvents. The results of this analysis are presented in Figure 9. It is interesting to note that all studied pairs of solvents exhibited positive deviations from the ideal mixing rule. This suggests that the interplay of solvent intermolecular interactions promotes solubility of phenacetin mixtures compared to pure solvents. All observed cosolvency effects were only weakly temperature-dependent, and only room temperature data is included in Figure 9. The strongest excess solubility was observed for 1,4-dioxane mixtures at *x*_2_* = 0.4. Conversely, methanol showed the smallest positive excess effect.

It is worth noting that the excess term is implemented in the Jouyban–Acree approach [64,65,66], which offers high flexibility of fitting by inclusion of a polynomial-type interpretation of excess solubility values. Although alternative formulations are often used to express concentrations in volume or mass solvents fractions, here concentration of mixed solvents is expressed in molar fractions. Hence, the excess solubility can be related to a Jouyban–Acree-type term according to the following formula:

(14) $$\Delta^{exc}ln\left(x_{1}^{bin}\left(T\right)\right)=x_{2}\cdot (1-x_{2})\overset{2}{\underset{i=0}{\sum }}\frac{J_{i}}{T}{\left(2x_{2}-1\right)}^{i}\cdot$$

**Figure 10:**
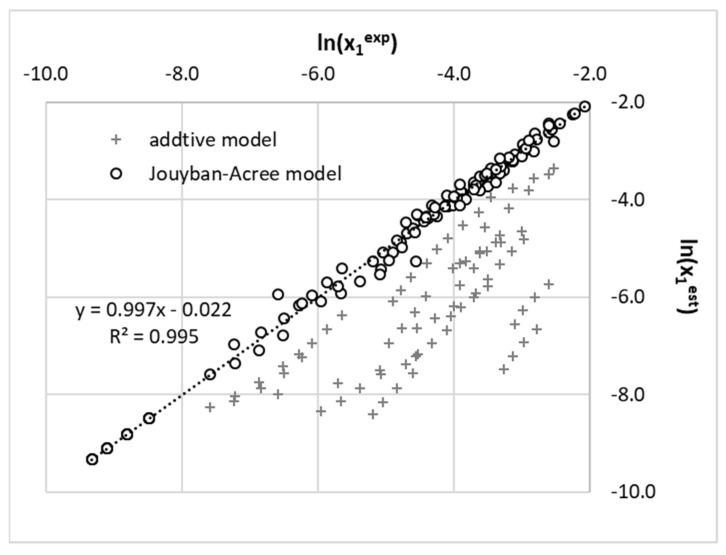
Comparison of accuracy of additive model (Equation (13)) and Jouyban–Acree approach defined in Equation (14).

The application of regression analysis leads to parameters summarized in Table 5, and the quality of fitting for the full set of 120 data points is presented in Figure 10. The strong non-ideal mixing can be directly inferred from the distribution shown in Figure 10.

## 3. Materials and Methods

### 3.1. Solubility Determination and Thermodynamic Measurements

#### 3.1.1. Chemicals

All solubility determination and calorimetric measurements were carried out using analytical grade chemicals. Phenacetin (CAS: 62-44-2) was obtained from Sigma-Aldrich (Poznań, Poland). The solvents acetonitrile (CAS: 75-05-08), dimethylformamide (DMF, CAS: 68-12-2), dimethyl sulfoxide (DMSO, CAS: 67-68-5), and methanol (CAS: 67-56-1) were provided by Avantor (Gliwice, Poland), while 1,4-dioxane (CAS: 123-91-1) was purchased from Sigma-Aldrich. Sodium chloride, potassium chloride, and salicylic acid used for heat capacity measurements validation were obtained from Avantor (Gliwice, Poland). The zinc melting standard (99.999%) used for the DSC calibration and the indium wire (99.999%) for calibration and heat capacity measurement validation were provided by the DSC device manufacturer (PerkinElmer, Waltham, MA, USA).

#### 3.1.2. Phenacetin Solubility Determination

In this study the shake-flask method was applied for solubility measurements. The procedure was based on methodology detailed in previous studies [25,67]. Briefly, the suspensions were prepared by adding 3 mL of the solvent to phenacetin in glass test tubes. The samples were then shaken at 60 rpm at constant temperature. The Orbital Shaker ES-20/60 (Biosan, Riga, Latvia) device was used for this purpose, providing uniform mixing and thermostating of the samples. After 24 h the mixing was stopped, and the samples were set for an hour to allow the sediment to settle. The saturated solution was then filtered using a syringe and 0.22 μm PTFE filter. A dilution of 0.1 mL of the filtrate with 2 mL of methanol was used for spectrophotometric measurements, while 0.5 mL of the filtrate was used for density determination of molar fraction solubility. Filtration and filtrate collection operations were performed in the shortest possible time, with all equipment (test tube, syringe, filter, and pipette tips) preheated to the solubility measurement temperature to avoid crystallization caused by temperature decrease.

Molar solubility was determined based on spectra measurements using a UV-VIS spectrophotometer (A360 AOE Instruments, Shanghai, China). Where absorbance was found to be too high, the samples were diluted with methanol accordingly to adjust to the linearity region of the calibration curve. The phenacetin concentration was determined from the calibration curve prepared using methanolic solutions (*λ*_max_ = 249 nm). The standard uncertainty of the solubility molar fraction, *u(x)* was estimated as 0.04.

#### 3.1.3. FTIR-ATR Analysis of Sediments

The phenacetin sediments obtained after solubility determination were dried and characterised using the FTIR method. For this purpose, an FTIR spectrum device (PerkinElmer, Waltham, MA, USA) with diamond Attenuated Total Reflection (ATR) accessory was used.

#### 3.1.4. Differential Scanning Calorimetry (DSC) Measurements

All DSC measurements were carried out by means of DSC 6000 calorimeter (Perkin Elmer, Waltham, MA, USA). The calibration was performed using zinc and indium standards. The nitrogen flow was set to 20 mL/min, while heat flow rate was 5 K/min in all cases except for the supercooling step. In order to obtain the experimental values of melting temperature (*T_m_*) and melting enthalpy (Δ*H_m_*), standard non-modulated thermograms were recorded. These parameters were determined based on analysis of the melting peak. The onset value was taken as the melting point, while the melting enthalpy was calculated based on the area under the peak. All calculations were performed automatically using Pyris software (PerkinElmer, Waltham, MA, USA). Non-modulated measurements were also applied to analysis of the sediments obtained after solubility determination. All non-modulated measurements were carried out using aluminium pans.

The heat capacity values of phenacetin in solid and supercooled liquid states were determined using the temperature-modulated DSC technique. In the case of the supercooled liquid heat capacity, measurements were carried out based on the methodology proposed by Rasmuson et al. [48,68,69,70,71]. To obtain the supercooled liquid state, the melted phenacetin was heated to 423 K, which is beyond the end of melting peak, and then it was rapidly cooled c.a. 10 K below the melting point to 398 K. The lack of crystallization peak during the cooling run confirmed that the phenacetin was in a supercooled state. Amplitude was set to 120 s during heat capacity measurement, while the modulation period was 1 K. The quality of the heat capacity determination was evaluated by the measurements performed for sodium chloride, potassium chloride, indium, and salicylic acid, and comparing the values obtained with literature data [68,72,73]. These results are summarized in supplementary materials (Figures S3–S6). With regard to three inorganic solids (NaCl, KCl, and In) the relative deviations from the reference values ranged from −1.5% to 2.5%. With salicylic acid, both solid and liquid states were considered, and the obtained *Cp*(*s*) = *f*(*T*) and *Cp*(*l*) = *f*(*T*) relationships appear to be quite close to the literature data [69]. All heat capacity measurements were performed using stainless steel large volume capsules dedicated to liquid samples. The standard uncertainties of thermodynamic properties determined using DSC measurements were estimated as *u*(*T_m_*) = 0.19, *u*(Δ*H_m_*) = 0.24, and *u*(*Cp*) = 0.02.

## 4. Conclusions

In this study, phenacetin solubility in six neat solvents (water, 1,4-dioxane, DMSO, DMF, acetonitrile, and methanol) and aqueous binary mixtures at four different temperatures were measured. It was noted that all these solvents offer a significant solubility advantage, enhancing phenacetin solubility by more than two orders of magnitude compared to water. Additionally, synergistic effects were noted in the case of aqueous solutions of acetonitrile and 1,4-dioxane. Following on from this, a sequence of solvents can be proposed, with decreasing values of solubility advantage in respect to water (T = 25 °C):

DMF (neat, SA = 2.92) > 1,4-dioxane (*x*_2_* = 0.6, SA = 2.70) > DMSO (neat, SA = 2.58)

> methanol (neat, SA = 2.34) > acetonitrile (*x*_2_* = 0.8, SA = 2.31)

> acetonitrile (neat, SA = 2.12) > 1,4-dioxane (neat, SA = 2.01)

where SA is expressed in logarithmic scale (SA = log(*x^cosolvent^*/*x^water^*)).

Additionally, the thermodynamic properties relevant to solubility modelling were measured by providing experimental detail of parameters such as melting point, enthalpy of fusion, and heat capacity of solid and melt states. The latter two properties allow for estimation of the values of heat capacity changes on melting and the direct characteristics of fusion thermodynamics. In the literature there are different approaches to such analysis, therefore detailed investigation was carried out on the influence of the ∆*Cp*(*T*) model on fusion thermodynamic functions, ideal solubility, and activity coefficients. The results suggested a surprising conclusion. It was observed that in the case of phenacetin, the complete omission of heat capacity change on melting in the mathematical formulas led to characteristics which are almost as accurate as complete inclusion of experimentally determined temperature relationship of ∆*Cp* values. This observation is contrary to common expectation and should be attributed to phenacetin rather than serving as a general trend. Comparisons of ideal solubility computed for some selected solids characterized by high and positive ∆*Cp* experimental values suggested that phenacetin is unique. For all the examples provided, the more restrictions imposed on the ∆*Cp*(*T*) model, the stronger the underestimation of ideal solubility. Hence, caution is advised before completely neglecting Δ*Cp*, since this may introduce significant errors to all related values such as fusion thermodynamics functions, ideal solubility, and consequently computed activity coefficients at saturated conditions. For further documentation relating to this concern, the data characterising errors of computed properties associated with different models of Δ*Cp*(*T*) were collected in Table 6. It can be clearly seen that the accuracies of some properties are quite acceptable for the crudest representation of Δ*Cp*, but this statement is far from a recommendation to ignore this important thermodynamic factor. Even model 2, which seems to be a milder version of model 1, is unacceptable for proper estimation of many properties. Hence it is possible to suggest that nothing is better than anything, at least if Δ*Cp*(*T*) is considered. This means that in the case of lack of experimental data of heat capacity, the values mimicking temperature trends with various simplifications can be misleading. This is obviously valid for phenacetin but might possibly be extended for other solids, provided that Δ*Cp* is small. As has been documented in the case of such compounds as myo-inositol, mannitol, risperidone, meglumine and many other solids with non-trivial contributions coming from heat capacities, the full temperature-related change of Δ*Cp*(*T*) should be included in the thermodynamic description.

## Figures and Tables

**Table 1:** Melting characteristics of phenacetin determined in this study and reported in the literature. The standard deviation values are provided in parentheses (*n* = 3).

Tm [K]	ΔHm [kJ/mol]
408.05 (±0.19) ^(1)^, 407.65 ^(2)^, 407.00 ^(3,4)^, 409.00 ^(5)^, 407.60 ^(6)^, 409.60 ^(7)^, 408.30 ^(8)^, 407.40 ^(9)^, 410.20 ^(10)^, 407.20 ^(11)^, 407.70 ^(12)^	32.45 (±0.24) ^(1)^, 30.72 ^(2)^, 28.79 ^(3)^, 32.00 ^(4)^, 31.50 ^(5)^, 36.93 ^(6)^, 30.00 ^(7)^, 28.75 ^(8)^, 34.10 ^(9)^, 21.40 ^(10)^, 31.25 ^(11)^, 32.33 ^(12)^

^(1)^ This study, ^(2)^ ref. [35], ^(3)^ ref. [39], ^(4)^ ref. [40], ^(5)^ ref. [41], ^(6)^ ref. [42], ^(7)^ ref. [43], ^(8)^ ref. [38], ^(9)^ ref. [44,45], ^(10)^ ref. [46], ^(11)^ ref. [47], ^(12)^ ref. [37].

**Table 2:** Summarized parameters of the models of heat capacity change upon melting used for the thermodynamic characteristics of solid and saturated solutions of phenacetin.

Model		*q* * [kJ·mol^−1^]	*r* * [kJ·mol^−1^·K^−1^]
1	experimentally derived linear trend $\Delta C_{p}=q+r\left(T_{m}-T\right)$	12.68	0.523
2	$\Delta C_{P}^{}\left(T\right)=\mathrm{const}\approx \Delta C_{p}^{m}=q$	12.68	zero
3	$\Delta C_{P}^{}\left(T\right)=\mathrm{const}\approx \frac{H_{P}^{m}\left(T=T_{m}^{}\right)}{T_{m}^{}}=\Delta S_{}^{m}$	79.52	zero
4	$\Delta C_{P}^{}\left(T\right)=\mathrm{const}=0.0$	zero	zero

* Melting temperature of this study was used.

**Table 3:** The solubility values of phenacetin expressed as molar fractions (×10^3^) along with the standard uncertainty (*n* = 3) determined in six neat solvents.

*T* [K]	298.15	303.15	308.15	313.15
water (10)	0.89 ± 0.05	1.11 ± 0.05	1.50 ± 0.05	2.07 ± 0.09
acetonitrile	11.81 ± 0.73	13.69 ± 0.28	16.10 ± 0.80	19.01 ± 0.45
1,4-dioxane	9.1 ± 0.5	12.4 ± 0.5	16.2 ± 0.6	20.3 ± 0.6
DMF	73.7 ± 1.6	86.9 ± 3.1	104.7 ± 3.3	124.6 ± 3.5
DMSO	34.1 ± 3.2	52.7 ± 2.7	77.2 ± 2.9	108.1 ± 5.0
methanol	19.6 ± 1.4	24.6 ± 1.2	31.6 ± 1.2	41.2 ± 1.1

**Table 4:** Collection of experimentally obtained values of phenacetin solubility in binary solvents along with the standard uncertainty (*n* = 3) expressed in mole fractions (×10^4^). First column characterizes concentration of organic solvent in solute free solutions (*x*_2_* denotes mole fraction of organic solvent in solute free binary aqueous mixture).

*x*_2_*	298.15	303.15	308.15	313.15
**acetonitrile + water**				
0.2	25.9 ± 1.0	34.5 ± 0.5	46.0 ± 1.7	62.8 ± 2.3
0.4	90.1 ± 2.9	107.3 ± 4.2	132.8 ± 4.6	165.4 ± 4.7
0.6	173.6 ± 5.0	202.9 ± 6.2	246.3 ± 2.9	300.3 ± 6.2
0.8	180.8 ± 3.0	216.7 ± 5.2	269.2 ± 5.4	336.2 ± 11.7
**1,4-dioxane + water**				
0.2	55.7 ± 2.3	64.2 ± 2.6	79.2 ± 1.9	99.9 ± 1.7
0.4	379.5 ± 12.7	433.2 ± 14.9	508.1 ± 7.0	616.1 ± 8.7
0.6	446.3 ± 13.0	504.2 ± 13.1	600.0 ± 8.3	739.8 ± 14.5
0.8	302.1 ± 7.9	358.0 ± 11.7	427.6 ± 9.7	510.7 ± 10.6
0.0	0.9 ± 0.0	1.1 ± 0.1	1.5 ± 0.0	2.1 ± 0.1
**DMF + water**				
0.2	13.8 ± 2.7	33.2 ± 2.1	61.1 ± 1.9	105.2 ± 3.0
0.4	106.6 ± 4.7	138.8 ± 4.7	183.4 ± 4.6	252.8 ± 4.5
0.6	199.3 ± 10.9	268.2 ± 8.8	364.7 ± 11.8	498.6 ± 9.7
0.8	315.7 ± 16.5	434.3 ± 24.7	591.2 ± 20.6	791.5 ± 14.8
**DMSO + water**				
0.2	7.2 ± 0.3	10.6 ± 0.5	15.0 ± 0.3	19.7 ± 0.7
0.4	70.5 ± 2.5	85.6 ± 1.1	102.7 ± 1.8	121.9 ± 2.7
0.6	200.5 ± 3.2	243.4 ± 8.8	297.6 ± 2.4	358.4 ± 6.6
0.8	290.2 ± 15.0	407.4 ± 19.7	552.7 ± 7.7	741.8 ± 15.9
**MeOH + water**				
0.2	5.1 ± 0.5	7.3 ± 0.6	10.4 ± 0.5	14.7 ± 0.5
0.4	18.8 ± 0.5	22.6 ± 0.9	28.3 ± 0.4	35.2 ± 1.0
0.6	74.6 ± 1.7	84.0 ± 3.5	97.4 ± 4.5	122.1 ± 1.1
0.8	142.6 ± 4.0	167.5 ± 5.5	210.0 ± 2.5	264.2 ± 6.5

**Table 5:** The parameters of Jouyban–Acree model defined in Equation (14) characterizing phenacetin solubility in aqueous organic binary mixtures. The last two columns characterize quality of fitting by providing values for root means square deviations (RMSD) and mean absolute percentage error (MAPE).

Cosolvent	*J*_0_ 10^3^	*J*_1_	*J*_2_ 10^3^	RMSD	MAPE
methanol	1.149	−12.40	0.759	0.10	1.23
1,4-dioxane	4.615	−1899.17	0.570	0.07	1.47
acetonitrile	3.063	−1511.42	1.836	0.07	1.15
DMF	2.309	−2257.29	0.989	0.22	2.85
DMSO	2.217	−168.14	−1.001	0.09	1.33

**Table 6:** Comparative analysis of accuracy of model accounting for heat capacity change upon melting. The mean values were computed in temperature range from *T* = 273.15 K to melting point. The relative difference is expressed as the ratio (*X*^(*i*)^ − *X*^(1)^)/X^(1)^⋅100%, where superscript denotes model of ∆*Cp* and *X* stands for one of the properties listed in the first column.

Property	Relative Difference			Mean Value			
	2 ÷ 1	3 ÷ 1	4 ÷ 1	1	2	3	4
∆Gfus [kJ/mol]	4.1%	−9.3%	6.6%	8.20	8.53	7.44	8.74
%H [%]	−1.1%	1.2%	−1.4%	58.6%	58.0%	59.3%	57.8%
x^id^	−12.6%	36.1%	−19.6%	0.036	0.032	0.050	0.029
ln(γ)[water]	−2.2%	5.1%	−3.6%	6.02	5.88	6.32	5.80
ln(γ)[methanol]	−21.3%	49.4%	−34.8%	0.62	0.49	0.93	0.41
ln(γ)[DMSO]	−188.2%	435.4%	−306.5%	0.07	−0.06	0.38	−0.15
ln(γ)[DMF]	19.0%	−43.9%	30.9%	−0.70	−0.83	−0.39	−0.92
ln(γ)[dioxane]	−9.6%	22.2%	−15.6%	1.39	1.25	1.69	1.17
ln(γ)[acetonitrile]	−11.8%	27.2%	−19.2%	1.13	1.00	1.44	0.91

## Data Availability

All data are available on request from the corresponding author.

## Supplementary Materials

The following are available online. A. Supplementary data documenting instrumental analysis: Figure S1. DSC thermograms recorded for solid precipitates obtained after shake-flask experiments; Figure S2. FTIR-ATR spectra recorded for solid precipitates obtained after shake-flask experiments; Figure S3. The results of DSC heat capacity measurements validation performed for NaCl; Figure S4. The results of DSC heat capacity measurements validation performed for KCl; Figure S5. The results of DSC heat capacity measurements validation performed for indium; Figure S6. The results of DSC heat capacity measurements validation performed for salicylic acid; B. Supplementary data documenting solubility measurements: Figure S7. The relationship between phenacetin solubility and binary solvent composition in acetonitrile-water; Figure S8. The relationship between phenacetin solubility and binary solvent composition in 1,4-dioxane-water; Figure S9. The relationship between phenacetin solubility and binary solvent composition in DMF-water; Figure S10. The relationship between phenacetin solubility and binary solvent composition in DMSO-water; Figure S11. The relationship between phenacetin solubility and binary solvent composition in methanol-water.

## Sample Availability

Samples are not available from the authors.
