# Supplementary material for: Thermodynamic Characteristics of Phenacetin in Solid State and Saturated Solutions in Several Neat and Binary Solvents
Source: Molecules. 2021 Jul 3;26(13):4078. doi: 10.3390/molecules26134078 (PMC8272242; doi:10.3390/molecules26134078)
Supplement: Supplementary file 1 [file molecules-26-04078-s001.zip › molecules-1260235-supplementary.pdf]

Supplementary Materials

Thermodynamic Characteristics of Phenacetin in Solid State and Saturated Solutions in Several Neat and Binary Solvents

Maciej Przybyłek[1], Anna Kowalska[1], Natalia Tymorek[1], Tomasz Dziaman[2] and Piotr Cysewski[1,*]

[1] Department of Physical Chemistry, Pharmacy Faculty, Collegium Medicum of Bydgoszcz, Nicolaus Copernicus University in Toruń, Kurpińskiego 5, 85-950 Bydgoszcz, Poland; m.przybylek@cm.umk (M.P.); 288310@stud.umk.pl (A.K.); 288369@stud.umk.pl (N.T.)
[2] Department of Clinical Biochemistry, Pharmacy Faculty, Collegium Medicum of Bydgoszcz, Nicolaus Copernicus University in Toruń, Karłowicza 24, 85-950 Bydgoszcz, Poland; tomekd@cm.umk.pl (T.D.)
* Correspondence: Piotr.Cysewski@cm.umk.pl

A. Supplementary data documenting instrumental analysis 2

Figure S1. DSC thermograms recorded for solid precipitates obtained after shake-flask experiments. 2

Figure S2. FTIR-ATR spectra recorded for solid precipitates obtained after shake-flask experiments. 2

Figure S3. The results of DSC heat capacity measurements validation performed for NaCl 3

Figure S4. The results of DSC heat capacity measurements validation performed for KCl 3

Figure S5. The results of DSC heat capacity measurements validation performed for indium 4

Figure S6. The results of DSC heat capacity measurements validation performed for salicylic acid. 4

B. Supplementary data documenting solubility measuremtns 5

Figure S7. The temperature and solvents ratio dependent solubility of Phenacetin in acetonitrile-water. The x_1 symbol stands for molar fraction solubility, while x_2^* denote organic component molar fraction in binary solvent. 5

Figure S8. The temperature and solvents ratio dependent solubility of Phenacetin in 1,4-dioxane-water. The x_1 symbol stands for molar fraction solubility, while x_2^* denote organic component molar fraction in binary solvent. 5

Figure S9. The temperature and solvents ratio dependent solubility of Phenacetin in DMF-water. The x_1 symbol stands for molar fraction solubility, while x_2^* denote organic component molar fraction in binary solvent. 6

Figure S10. The temperature and solvents ratio dependent solubility of Phenacetin in DMSO-water. The x_1 symbol stands for molar fraction solubility, while x_2^* denote organic component molar fraction in binary solvent. 6

Figure S11. The temperature and solvents ratio dependent solubility of Phenacetin in methanol-water. The x_1 symbol stands for molar fraction solubility, while x_2^* denote organic component molar fraction in binary solvent. 7

A. Supplementary data documenting instrumental analysis

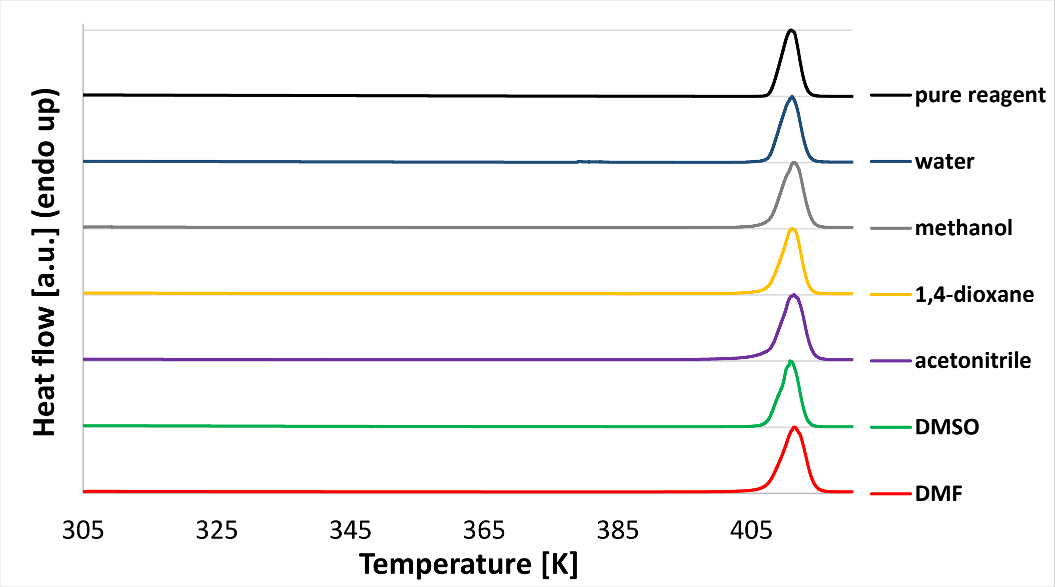

Figure S1. DSC thermograms recorded for solid precipitates obtained after shake-flask experiments.

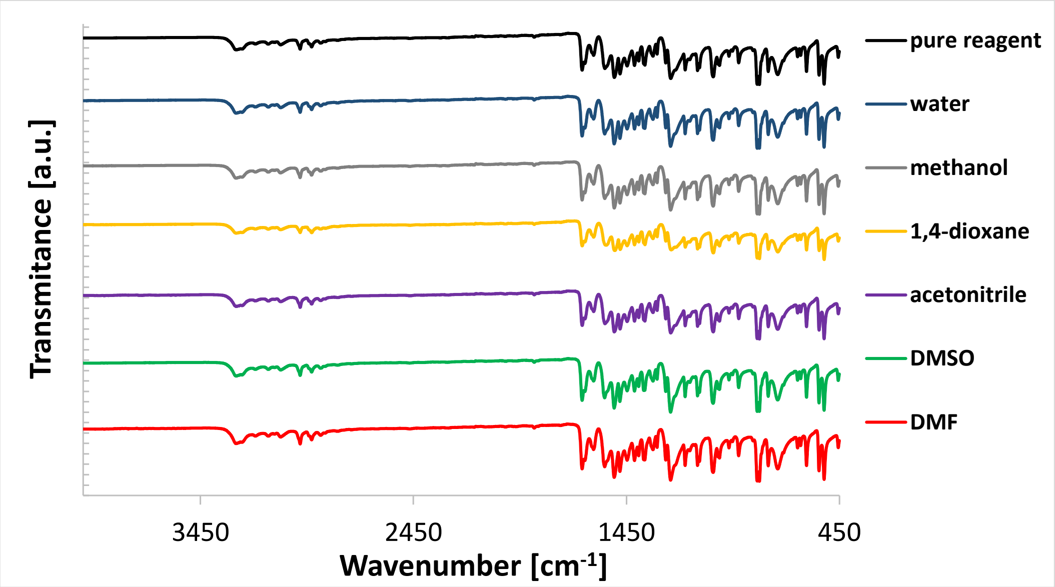

Figure S2. FTIR-ATR spectra recorded for solid precipitates obtained after shake-flask experiments.

solid	T [K]	this work	Ref.[68]	% deviation
NaCl	300	49.61	50.54	1.8%
	400	51.08	52.39	2.5%
	500	52.83	53.9	2.0%
	600	54.42	55.46	1.9%

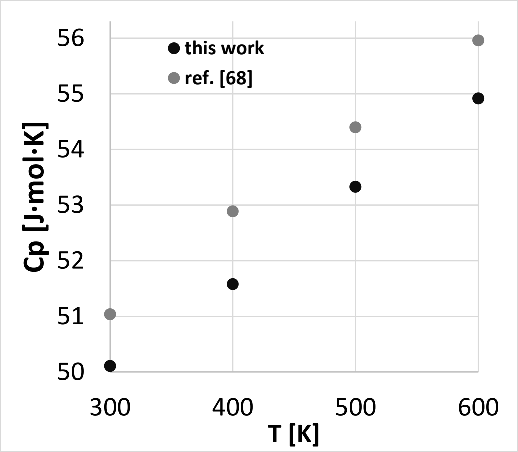

Figure S3. The results of DSC heat capacity measurements validation performed for NaCl.

solid	T [K]	this work	Ref.[68]	% deviation
KCl	300	52.09	51.33	-1.5%
	400	53.63	53.13	-0.9%
	500	55.23	54.77	-0.8%
	600	56.9	56.31	-1.0%

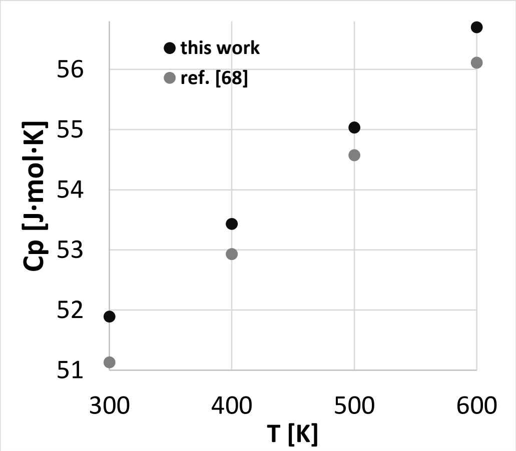

Figure S4. The results of DSC heat capacity measurements validation performed for KCl.

solid	T [K]	this work	Ref.[70]	% deviation
Indium	300	26.84	26.93	0.3%
	350	27.51	27.87	1.3%
	400	28.32	28.85	1.8%

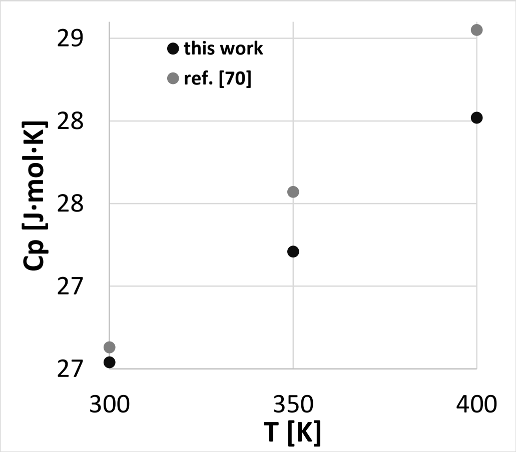

Figure S5. The results of DSC heat capacity measurements validation performed for indium.

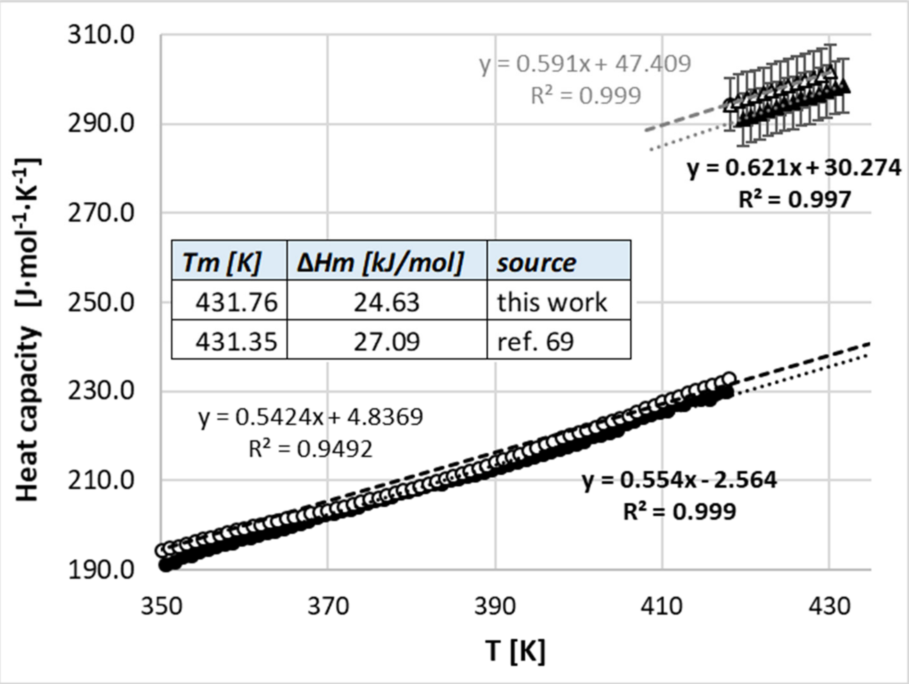

Figure S6. The results of DSC heat capacity measurements validation performed for salicylic acid.Black circles denote results obtained in this work, while the gray ones correspond to the literature data from ref. [69]. The error bars mark 2.0% accuracy of values obtained for melts.

B. Supplementary data documenting solubility measuremtns

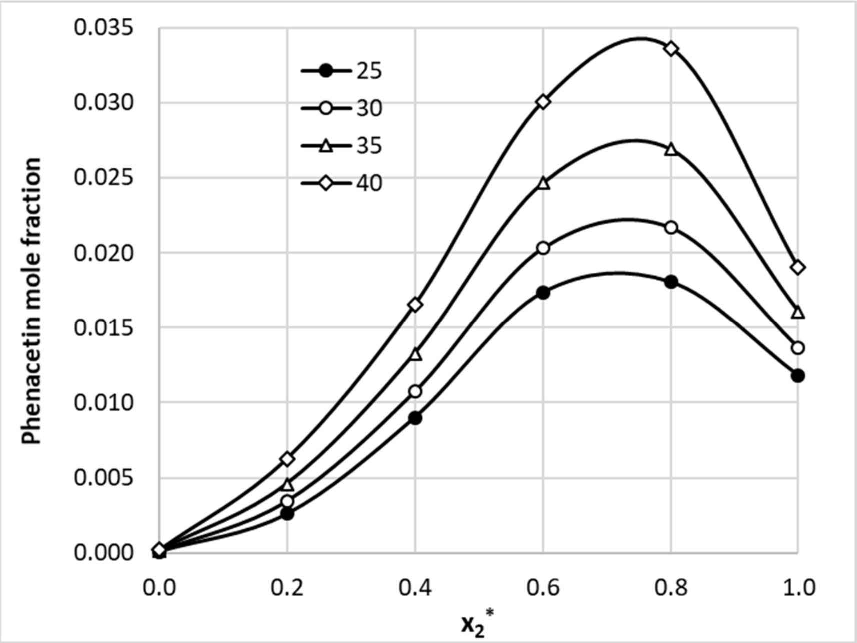

Figure S7. The temperature and solvents ratio dependent solubility of Phenacetin in acetonitrile-water. The x_1 symbol stands for molar fraction solubility, while x_2^* denote organic component molar fraction in binary solvent.

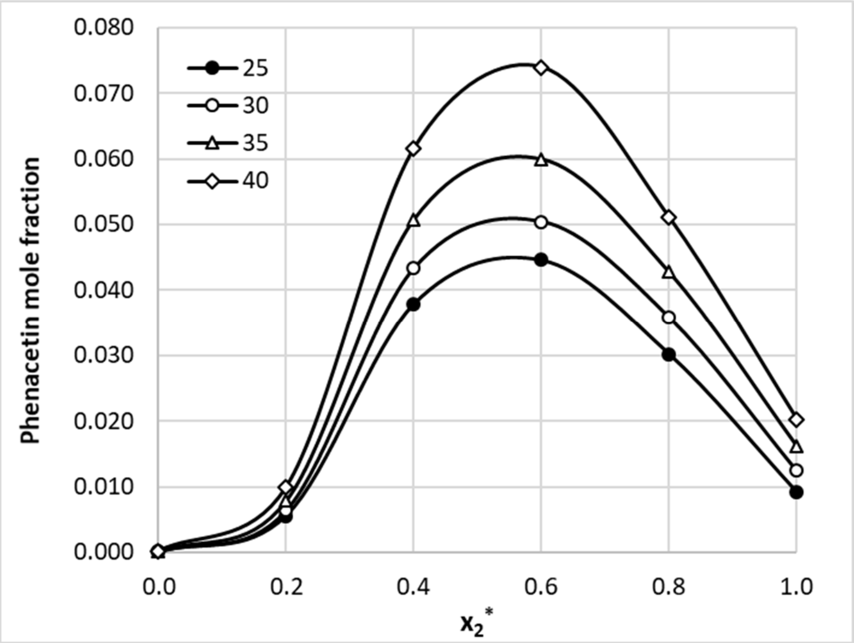

Figure S8. The temperature and solvents ratio dependent solubility of Phenacetin in 1,4-dioxane-water. The x_1 symbol stands for molar fraction solubility, while x_2^* denote organic component molar fraction in binary solvent.

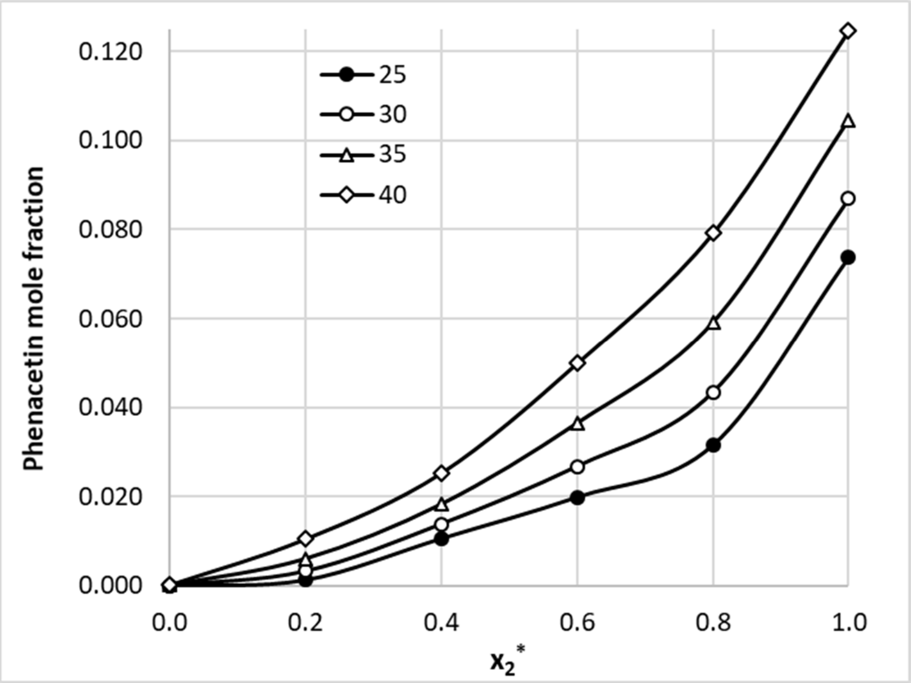

Figure S9. The temperature and solvents ratio dependent solubility of Phenacetin in DMF-water. The x_1 symbol stands for molar fraction solubility, while x_2^* denote organic component molar fraction in binary solvent.

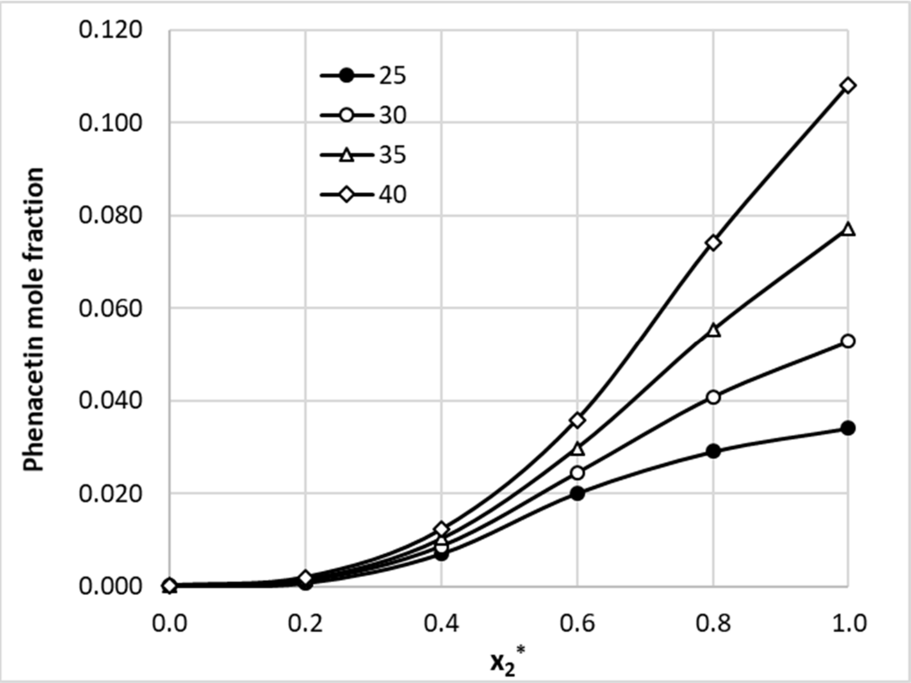

Figure S10. The temperature and solvents ratio dependent solubility of Phenacetin in DMSO-water. The x_1 symbol stands for molar fraction solubility, while x_2^* denote organic component molar fraction in binary solvent.

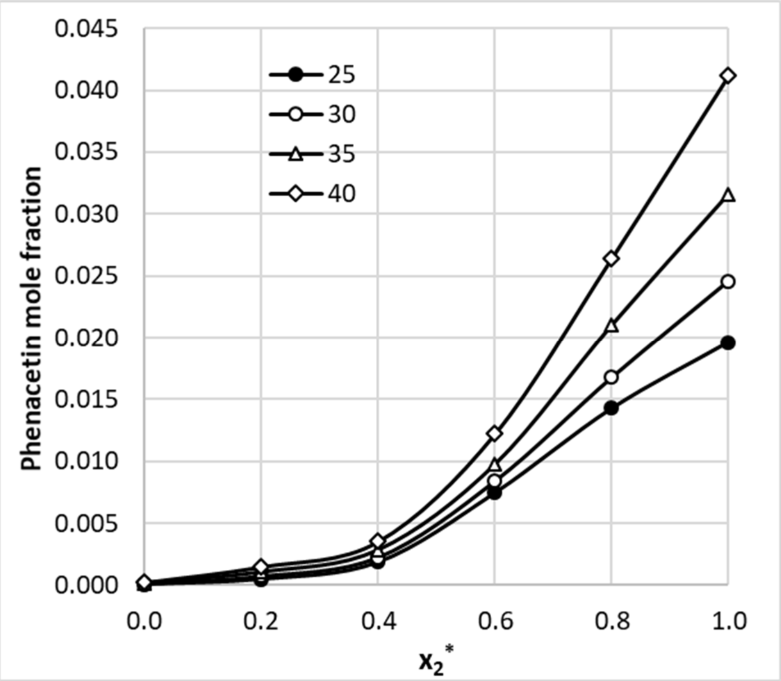

Figure S11. The temperature and solvents ratio dependent solubility of Phenacetin in methanol-water. The x_1 symbol stands for molar fraction solubility, while x_2^* denote organic component molar fraction in binary solvent.
